# Endemic malaria: an 'indoor' disease in northern Europe. Historical data analysed

**DOI:** 10.1186/1475-2875-4-19

**Published:** 2005-04-25

**Authors:** Lena Huldén, Larry Huldén, Kari Heliövaara

**Affiliations:** 1 Department of Applied Biology, Faculty of Agriculture and Forestry, University of Helsinki, Finland; 2 Finnish Museum of Natural History, University of Helsinki, Finland

## Abstract

**Background:**

Endemic northern malaria reached 68°N latitude in Europe during the 19^th ^century, where the summer mean temperature only irregularly exceeded 16°C, the lower limit needed for sporogony of *Plasmodium vivax*. Because of the available historical material and little use of quinine, Finland was suitable for an analysis of endemic malaria and temperature.

**Methods:**

Annual malaria death frequencies during 1800–1870 extracted from parish records were analysed against long-term temperature records in Finland, Russia and Sweden. Supporting data from 1750–1799 were used in the interpretation of the results. The life cycle and behaviour of the anopheline mosquitoes were interpreted according to the literature.

**Results:**

Malaria frequencies correlated strongly with the mean temperature of June and July of the preceding summer, corresponding to larval development of the vector. Hatching of imagoes peaks in the middle of August, when the temperature most years is too low for the sporogony of *Plasmodium*. After mating some of the females hibernate in human dwellings. If the female gets gametocytes from infective humans, the development of *Plasmodium *can only continue indoors, in heated buildings.

**Conclusion:**

Northern malaria existed in a cold climate by means of summer dormancy of hypnozoites in humans and indoor transmission of sporozoites throughout the winter by semiactive hibernating mosquitoes. Variable climatic conditions did not affect this relationship. The epidemics, however, were regulated by the population size of the mosquitoes which, in turn, ultimately was controlled by the temperatures of the preceding summer.

## Background

Endemic malaria was declining in western Europe from the 18^th ^century onwards, but in the 19^th ^century it was still common in the north and north-east of Europe. In the 1930s, endemic malaria finally disappeared from most parts of Europe, with the exception of occasional epidemics during the Second World War [1]. There is very little modern research on the historical conditions of the disease or even its historical distribution. Most published studies of historical distribution do not include the cases in Finland [2]. Imported cases during the Second World War, however, have attracted some attention [3].

Northern malaria has usually been connected with *Plasmodium vivax*. After infection, the sporozoites enter human hepatocytes and develop into hypnozoites. These remain in dormancy for a variable time, and the patient may occasionally suffer from malaria for up to four years and have symptoms every second month [4]. Sokolova & Snow [5] report that *Plasmodium falciparum *occurred in Archangelsk and Vologda in the northern part of the Soviet Union in the 1930s. It is usually assumed that *P. falciparum *lacks a dormant stage. In the Finnish medical reports the symptoms of malaria included tertian, quotidian and quartan malaria. The tertian form was the most common and Sievers [6] connected this form with *P. vivax*. The possible historical occurrence of other *Plasmodium *species in Finland remains uncertain.

The temperatures needed for the sporogony have been studied in laboratory conditions [4]. Garnham [7,8] limits the range of malaria to summer temperatures. The northern range of the occurrences of endemic malaria has been estimated to coincide with a summer isotherm of 16°C. However, even a superficial examination shows that malaria also occurred in the northern parts of Sweden and Finland, where the summer temperature was considerably below 16°C.

The present study aims to establish that malaria can spread in a cold climate, even when the outside temperature is lower than needed for the sporogony of the *Plasmodium*. Because of the high quality of available historical sources, of demographic, medical and temperature data, Finland is a very suitable area for the study. The study focused on the period 1800–1870, since the only effective drug, quinine, was not then frequently used by the common people. Its wide use would have biased the statistics of the causes of death. High resolution temperature data are also available for that period.

## Methods

The known distribution of three *Anopheles *species in Finland was published by Utrio [9]. The distribution unit of 50 × 50 km^2 ^gives a reasonably good resolution of the distribution of these species in Finland. Additional information from Sweden and Estonia was also used for comparison [10,11]. Life cycles, behaviour and the phenology of the mosquito has been interpreted according to the literature [12-18] and field samplings in the south-west archipelago by the first author. The data of historical malaria cases were collected and tested by historical methods. Although the historical data can be questioned, it should be emphasized that the Finnish sources give a good picture of the actual situation, and that the statistics are of a better quality than in most other countries. Causes of death were especially recorded by ministers in hundreds of parishes and, although there probably are some mistakes, most of the entries were correct.

There are two different kinds of source for collecting the records of malaria cases in Finland during the years 1800–1870: 1) reports by the district physicians and 2) the causes of death that were recorded by the local ministers. The physicians wrote an annual report to the medical board in Helsinki about the health conditions in their district. At the end of the century a separate report on epidemics was added. Malaria is mentioned in both kinds of reports. The reports had a free format and the number of sick patients was only rarely mentioned. Usually the physician formulated an opinion of the severity of the actual epidemics. Many districts were so large that the physician could hardly know the actual situation in the more remote parts, especially in the north. The archival series of physicians' reports include reports from the beginning of the 19^th ^century. The oldest reports contained very general information. They improved in 1857 when the number of districts increased to 50 [19]. Malaria was mentioned in 542 reports between 1826 and 1870. Sievers [6], who studied malaria in Finland, based his opinion solely on these reports. Sievers' interpretation was compared with the original documents, that can be examined in the National Archives in Helsinki, Finland. The reports cannot be used for quantitative analyses, but they mention the years in which they were epidemics and these coincide with the epidemic years in the death statistics.

From 1749, the minister had to record the cause of death for every diseased parish member in the burial records. The parish burial registers can therefore be used for detailed statistics on malaria. For this study these local records were used. The minister often attended the deathbed and, in contrast to the district physician, actually saw the patient. Ministers were probably very familiar with the typical symptoms of malaria, as it was a common disease. They often had some medical knowledge because the training in medicine for students of theology had developed rapidly duringthe second half of the 18^th ^century. The minister was not only responsible for the spiritual guidance of his parish members, but also for their bodily welfare [20]. In the present study, only those cases which the ministers recorded as malaria (5,431 death cases) have been taken into account. In the parish records, several words were used for malaria. *Frossa, fråssa, frossfeber *were the most common and other frequently used words were *kallfeber*, *kallsot*, *kylfeber*, *skälvan*, *skälvasot*, *tertian*, *tredjedagsfrossa*, *vardagsfrossa*, *skärgårdsfeber*, *växelfeber*, *omväxlingsfeber*, *intermittent feber *and *malaria*. The district physicians used *frossa*, *tredjedagsfrossa*, *vardagsfrossa*, *tertian*, *quartana *or *quotidiana*. These words were used in both Swedish and Finnish medical research from the 18th to the 20th century along with *skärgårdsfeber *[6,20-27]. The use of the above-mentioned words was strictly separated from the use of other words for diseases, which included fever or resembled malaria like *hetsig feber*, *flussfeber*, *feber*, *scharlakansfeber*, *älta *and *förkylning*. The consistent use of these malaria words is also confirmed by comparative correlation analyses. The statistics for cause of death is most representative for the period of 1800–1850. The data are collected from digitalized parish records, which were made available on the Internet by the Finnish Genealogical Society through Dec. 31^st^, 2003 [28]. The project is not yet finished, but most of the material before 1850 has been digitalized. The period of 1850–1900 is not very representative and only material from a few parishes is included. In the present study all cases recorded in southern Finland until 1870 are included but the last decade is under-represented in the material. It must be stressed that the records only show the number of deaths due to malaria but that the relation between the number of infected and the death rate is uncertain.

**Figure 1:**
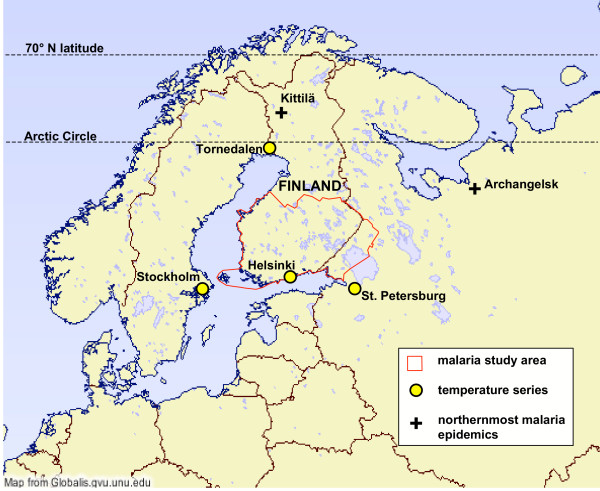
Map of malaria study area and locations of temperature series.

The longest Finnish temperature record from Helsinki, starting from 1829, is used as the basis for climatic correlations for the years 1829–1870. Temperatures from St. Petersburg (Russia) (1805–1870), Tornedalen (Sweden)(1818–1870) and Stockholm (Sweden)(1800–1870) are used as supporting data for the extended period of 1800–1870 (Figure 1). These are the best high resolution temperature data available at the present time [29-32].

## Results

### The distribution and life cycle of anophelines in Finland

Three *Anopheles *species have been reported from Finland [9]. *Anopheles beklemishevi *has a northern distribution in Finland, while the other common species, *Anopheles messeae*, is dominant in the southern part of the country [17]. *Anopheles claviger *is recorded from the Aland Islands and was also found by the authors in the south-western archipelago. In December 2004 and January 2005 flying specimens of an unidentified *Anopheles *(cf. *messeae*) were observed in summer cottages in two different localities in southern Finland.

Because of many taxonomical problems in the *Anopheles *genus, there is conflicting information on the details of the life cycles of the separate species. A general feature of the northern species is that the adult female hibernates. There are possibilities of a summer generation, at least in warm summers as in 1901 in Finland (possibly *A. messeae*)[9,12]. *A. claviger *hibernates as larvae [33] and, at least in England, seems to be bivoltine [34]. In Finland, the majority of the hibernating females reach the adult stage in the middle of August. The male is short lived and dies soon after mating. The female may suck blood or honey-dew before seeking shelter in sheds and houses for hibernation. Because of the cold climate the female cannot leave the hibernation site before spring. In the traditional agricultural society of Finland many of the mosquito females spent the main part of their adult life together with man and domestic animals. In warm conditions the female may take several blood meals during the hibernation, but it will not lay eggs before the spring [13].

*A. messeae *has been considered an important vector of historical endemic malaria in several parts of Europe and was also an important vector in the Soviet Union [4,15]. In the northern parts of Russia and along the Baltic coast, *A. beklemishevi *was an important vector [35]. *A. claviger *has also locally been reported as a vector [15]. Some recent research, however, has questioned *A. messeae *as a vector of malaria [36]. Jaenson & Ameneshewa [37] reported that blood-feeding was not a prerequisite for hibernation of *A. messeae*. This does not exclude the possibility of repeated indoor blood-feeding in warm indoor conditions. There is still unresolved and conflicting information on the feeding, resting and hibernation habits of the female (exophagy or endophagy, zoophily or anthropophily, exophily or endophily, complete diapause or semiactive winter habit). The anophelines in the northern region, however, must be flexible in their habits to survive the strong annual and seasonal fluctuations in temperature (-51°C to +33°C in Finland and rain/snow). In any case, one or more species, distributed over the whole of Finland, have been vectors of malaria in Finland. Yet it is likely that one or more additional species, still not reported from Finland, will be found as four additional species have been reported from Estonia and Sweden [10,11]. For the time being it is not possible to define which mosquito species was important for the malaria transmission in Finland.

### Endemic malaria in Finland

The *Anopheles *species, which have been vectors of malaria in Finland, presumably have existed here since prehistoric times. It is, however, probable that malaria reached Finland only during the 17^th ^century. There are several cases of malaria in the Mälaren region to the west of Stockholm in Sweden during that time. Both 1692 and 1693 are reported as years of severe fever [38], which spread rapidly also to the east. A total of 1,803 persons died of malaria in the western parts of Finland and in the south-western archipelago during the years 1751–1773 [23]. Haartman [21] reports severe epidemics in the region of Turku in the years 1774–1777 and the physician F.W. Radloff mentioned that malaria was very common in the Aland Islands in 1795 [39].

From 1800 to 1870, there were at least 5,431 death from malaria in Finland. During this period the Finnish population grew from 832,700 to 2,032,700 people [40]. In a few epidemics, the mortality can be estimated to 0.85–3.0% [6]. The number of deaths from malaria increased when the number of malaria cases increased and thus a change in virulence of the *Plasmodium *is not to be assumed. The total number of malaria cases in the middle of the 19^th ^century may tentatively be estimated to be 100,000–300,000 or about 7–20% of the whole population.

Although the source value of both the death statistics and the medical reports can be discussed separately, they are strengthened when compared with each other. As sources they are completely independent, and they partly overlap the period 1850–1870, showing similar trends. The district physicians reported 174 local epidemics during 1854–1862 and during the same period the deaths from malaria rose to 1,687 cases. In the years 1873–74 and 1877, the number of epidemics also rose and the same trend can be seen in the parish death records.

### The malaria epidemics and temperature

The worst malaria year in Finland was 1862. The eastern parts of Finland were particularly affected, including Karelia. Several physician's reports reveal how severe the situation was. In the district of Mikkeli over 4,000 persons became ill. Moreover, in the district of Joensuu 4,000 persons suffered, in Rautalampi several thousands and in the worst parishes in the districts of Viipuri and Muola every third person became ill. In eastern Finland the summer was cold and damp. Malaria epidemics even broke out in the north and in Sotkamo in the Kajana district more than a hundred people became ill.

**Figure 2:**
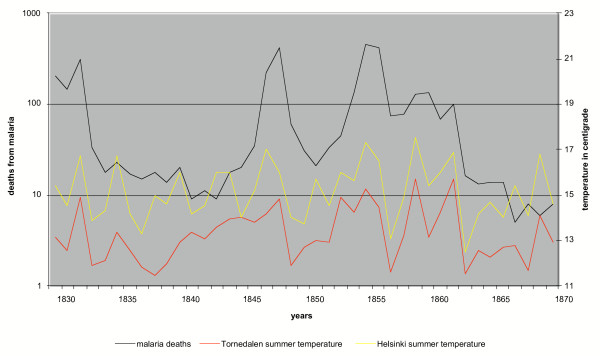
Malaria deaths in southern Finland and mean temperatures of the preceding summer in Helsinki and Tornedalen 1830–1870.

The correlation between temperature and the number of malaria deaths was tested on annual, seasonal and monthly levels. The seasons were interpreted as follows: winter (DJF), spring (MAM), summer (JJA) and autumn (SON). The malaria cases were tested against the temperatures of both the current malaria year and the preceding year. In Table 4 the calculations of monthly correlations were repeated with a square root transformation of the number of malaria cases to avoid biases caused by strong peaks during epidemics. Malaria deaths peak late in the spring but for completeness the calculations are extended further to the autumn of the malaria year. The results are presented in Tables 1,2,3,4 and Figure 2.

**Table 1:** Annual mean temperature correlated with malaria cases in 1800–1870.

Year correlated with malaria cases in 1800–1870	Source of temperature series			
	
	Helsinki 1830–1870 41 years	Tornedalen 1818–1870 53 years	Stockholm 1800–1870 71 years	St. Petersburg 1806–1870 65 years
malaria year-1	0.1364	0.1133	0.1238	0.0958
malaria year	0.0996	-0.1177	-0.0822	-0.1164

**Table 2:** Seasonal mean temperature correlated with malaria cases in 1800–1870

Season correlated with malaria cases in 1800–1870	Source of temperature series			
	
	Helsinki 1830–1870 41 years	Tornedalen 1818–1870 53 years	Stockholm 1800–1870 71 years	St. Petersburg 1806–1870 65 years
malaria year-1; winter	-0.1932	-0.1209	-0.1652	-0.0789
malaria year-1; spring	-0.0091	-0.0228	0.0248	-0.0312
**malaria year-1; summer**	0.5120 ***	0.5208 ***	0.4708 ***	0.3981 ***
malaria year-1; autumn	0.1218	0.1621	0.1055	0.1598
malaria year; winter	-0.1254	-0.0743	-0.1214	-0.1543
malaria year; spring	0.1140	0.0880	0.0223	0.0546
malaria year; summer	-0.0740	-0.0332	0.0201	-0.0439
malaria year; autumn	-0.0831	-0.0819	-0.0211	-0.0797

**Table 3:** Monthly mean temperature correlated with malaria cases in 1800–1870.

Month of preceding year correlated with malaria cases in 1800–1870	Source of temperature series			
	
	Helsinki 1830–1870 41 years	Tornedalen 1818–1870 53 years	Stockholm 1800–1870 71 years	St. Petersburg 1806–1870 65 years
malaria year-1; May	0.1487	0.0269	0.0511	0.1338
**malaria year-1; June**	0.4301 **	0.5289 ***	0.3372 **	0.3587 **
**malaria year-1; July**	0.4628 **	0.4135 **	0.4543 ***	0.2888 *
malaria year-1; August	0.2672°	0.1570	0.2740 *	0.1793
malaria year-1; September	0.1156	0.0655	0.0637	0.0678
malaria year-1; October	0.1566	0.0200	0.1326	0.2657 *
malaria year-1; November	0.1462	0.1958	0.0000	0.0220
malaria year-1; December	0.0037	-0.002	-0.1144	-0.1221

**Table 4:** Monthly mean temperature correlated with square root transformed malaria cases in 1800–1870

Month of preceding year correlated with square root transformed malaria cases in 1800–1870	Source of temperature series			
	
	Helsinki 1830–1870 41 years	Tornedalen 1818–1870 53 years	Stockholm 1800–1870 71 years	St. Petersburg 1806–1870 65 years
malaria year-1; May	0.1512	0.0770	0.0623	0.1389
**malaria year-1; June**	0.4407 **	0.5739 ***	0.4049 ***	0.3780 **
**malaria year-1; July**	0.4801 **	0.4511 ***	0.5081 ***	0.3275 **
malaria year-1; August	0.2428	0.1174	0.2610 *	0.1657
malaria year-1; September	-0.1479	0.0469	0.0824	0.0280
malaria year-1; October	0.1591	0.0322	0.1487	0,2145°
malaria year-1; November	0.1428	0.2048	0.0039	0.0137
malaria year-1; December	0.0190	0.0055	-0.1049	-0.1250

Annual mean temperatures were obviously not significant in the correlations. On the seasonal level the summer of the preceding year is highly significant (0.1% risk level). In all other seasons the risk level was more than 10%. Renkonen [26] tried to show a correlation between malaria epidemics and the temperature of April or spring. The correlation coefficient for both April and the entire spring were close to zero and thus Renkonen's hypothesis can be rejected.

June and July of the preceding summer were very significant with a faint "tail" in August. The weak correlation of October temperatures in the St. Petersburg series (5% and 10% risk level respectively in Tables 3,4) can safely be considered stochastic noise with no support from the other series.

Death caused by what were called 'intense fever', *hetsig feber *(48853 cases), cold, influenza, *flussfeber *(7670 cases), fever, *feber *(64604 cases) and *cold*, *förkylning *(1381 cases) were tested for correlations with both temperatures and with malaria in 1800–1850, but no correlations were found. The words expected to indicate malaria are well-defined and separated from other kind of fevers and they can safely be analysed as a homogeneous disease.

The correlation is strongest with the season when the vector is in the larval stage. Thus the number of malaria cases correlates with the warmest time of the summer when there is no *Plasmodium *present in connection with the mosquito. The conclusion is that fluctuations in summer temperatures regulate the number of mosquitoes reaching the adult stage and not the development of *Plasmodium*. Because malaria epidemics covariate with the summer temperatures, the sporogony inside the mosquito may be treated as a constant parameter. Thus the sporogony of *Plasmodium *in Finland must have taken place in reasonably stable temperature conditions.

**Figure 3:**
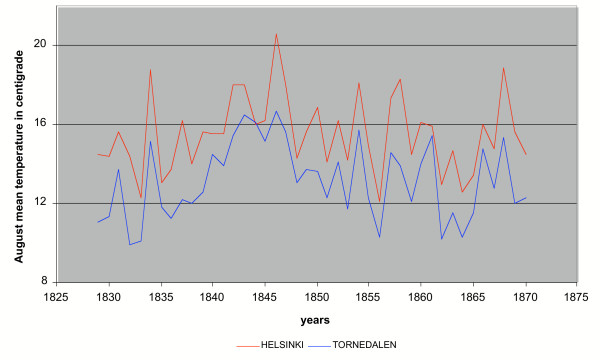
Mean temperature of August in Helsinki and Tornedalen 1829–1870.

The sporogony is temperature dependant and the duration of the sporogony of *P. vivax *is 7–8 days in 30°C, 8–10 days in 28°C, 15–16 days in 20–21°C and 55 days near 16°C with development stopping completely below 16°C [4]. When the mosquito reached the adult stage in August, the temperature was already falling. If the mosquito took a blood meal from a carrier of malaria late in the summer or at the beginning of autumn, the malaria parasite would have had no time to complete its sporogony in the vector in outside conditions. August was the warmest month the adult mosquito female encountered during its life. The mean temperatures of August and May during the years 1829–1870 were in Helsinki 15.53°C (range 12.3–20.6°C) and 7.34°C respectively and the corresponding temperatures in Tornedalen 13.15°C (range 9.9–16.7°C) and 4.53°C. These values give a realistic picture of the temperature conditions within the area where endemic malaria occurred in Finland. The summer season in Finland was within a wide range clearly unsuitable for the development of *Plasmodium *(Figure 3).

The principal explanation for continuation of endemic malaria must be the choice of hibernation shelter by the female mosquito, within a warm environment in close proximity to humans. Such places in previous centuries were animal shelters and human dwellings. An indoor transmission cycle was first suggested by Robert Koch in the 1890's [25] and Swellengrebel and de Buck [41] gave a detailed description of indoor infection by hibernating mosquitoes in the Netherlands. The situation in Finland was different. Sivén [25] noted during the epidemic in Helsinki in 1902, that many infants who got malaria in the spring, were born in the winter. In the years 1750–1850 there were about 40–50 corresponding death cases. As a consequence hibernating mosquito females were active and capable of infecting humans during the whole winter.

**Figure 4:**
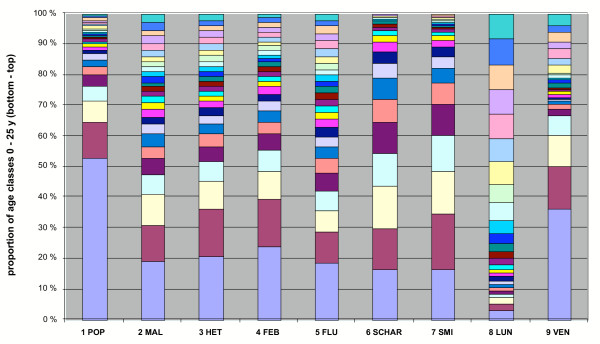
Relative proportions of annual age classes (of deaths) of the total population, malaria and seven other diseases in 1750–1850 (110,538 cases without age data was excluded), in the 0–25 year age group. The number of cases are as follows: (1) total population 1,143,547; (2) malaria 3,128; (3) hetsig feber (intense fever) 38,368; (4) feber (fever) 32,426; (5) flussfeber (cold, influenza) 4,681; (6) scharlakansfeber (scarlet fever) 10,202; (7) smittkoppor (smallpox) 100,768; (8) lungsot (phthisis) 13,674; (9) venerisk sjukdom (venereal disease) 462

**Figure 5:**
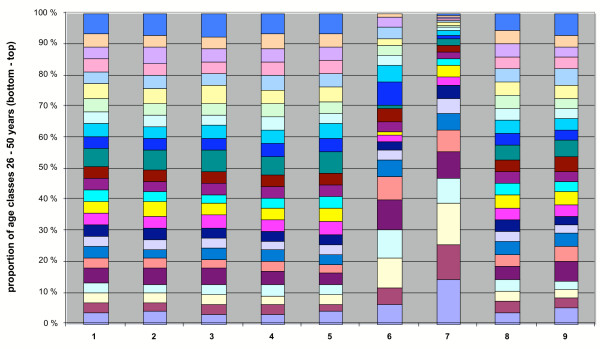
Relative proportions of annual age classes (of deaths) of the total population, malaria and seven other diseases in 1750–1850, in the 26–50 year age group. The number of cases are as follows: (1) total population 238,047; (2) malaria 2,554; (3) hetsig feber (intense fever) 22,325; (4) feber (fever) 16,038; (5) flussfeber (cold, influenza) 3,690; (6) scharlakansfeber (scarlet fever) 95; (7) smittkoppor (smallpox) 902; (8) lungsot (phthisis) 33,950; (9) venerisk sjukdom (venereal disease) 364

**Figure 6:**
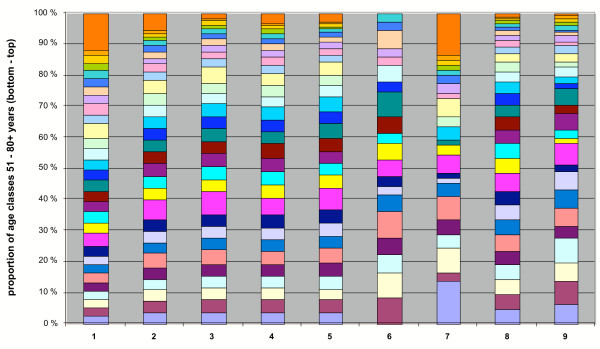
Relative proportions of annual age classes (of deaths) of the total population, malaria and seven other diseases in 1750–1850, in the 51–80+ year age group. The number of cases are as follows: (1) total population 413,775; (2) malaria 3,364; (3) hetsig feber (intense fever) 25,560; (4) feber (fever) 21,816; (5) flussfeber (cold, influenza) 4,773; (6) scharlakansfeber (scarlet fever) 36; (7) smittkoppor (smallpox) 66; (8) lungsot (phthisis) 31,122; (9) venerisk sjukdom (venereal disease) 172

The relative proportion of annual deaths in different age groups of the total population was compared in Figures 4,5,6, for malaria and seven other diseases in the years 1750–1850. The age group structure of malaria deaths is very similar to some other fevers and approaches that of the total population. Deviations in the total population are mainly explained by increased (>50%) omissions of the cause of death in children under two years old in the parish records. Malaria seems to spread with equal probability among all age groups. If the majority of infections had occurred during a limited time in late summer or the beginning of autumn, the resulting age group distribution of malaria deaths would have been different. In the traditional agricultural society, certain age groups used to live apart during the summer, usually in unheated buildings. In October-November, decreasing temperatures forced them to move back into heated buildings [42]. Thus, the most conservative explanation is an extended cold season infection time when all age groups congregate in heated buildings.

Hernberg [43,44] studied the malaria epidemic in Finland during the years 1941–1945, resulting from infections acquired during the war. He concluded that *P. vivax *has a variable incubation time of 5–12 months or more. Another conclusion was, that the spring peak of malaria was a result of an infection in the previous year, because he assumed that no mosquitoes were present during the winter. He obviously did not note Sivén's observation of winter infections. Winter infections were probably the reason why Renkonen tried to correlate the infections with the mean temperature of March [26].

According to Swellengrebel and de Buck [41], the main infection time of malaria in the Netherlands was from the middle of August to the middle of November and there were practically no infections after November because of degenerating sporozoites. The degeneration of sporozoites late in the autumn was due to low temperature in unheated bedrooms. In Finland, however, hibernating mosquitoes have been able to infect humans during the whole winter because of heated bedrooms. The importance of the dormancy of the *Plasmodium *is pronounced in the summer, as the parasite needs a prolonged dormant stage in humans to survive the summer. The summer temperature conditions are unstable and highly variable and there are no or too few anopheline females during a considerable part of the warm season. The winter temperature conditions are stable inside human dwellings and the mosquito female can repeatedly produce sporozoites and infect humans. Some of the sporozoites immediately initiate schizogony while most of them remain as dormant hypnozoites in the hepatocytes. Another source for schizonts may be gradual activation of dormant hypnozoites from the preceding winter. As a consequence, during the erythrocytic cycle some gametocytes are produced throughout the winter in high latitudes. It has been suggested that hormonal changes in the host, controlled by the changing light-cycle, induce mass activation of the hypnozoites in the spring [45]. The spring peak of hypnozoites could be of no importance for the *Plasmodium *in Finland. Some of the hypnozoites, however, were activated only in the following autumn or later. Thus, paradoxically, summer dormancy was crucial for maintaining the *Plasmodium *until the next generation of anopheline females were present. Such a pattern had already been suggested, in parts, by Reiter [1].

## Discussion

Endemic malaria in Finland was independent of outdoor temperature conditions. The infection could not spread in Finland during the summer because possible summer generations of mosquitoes are irregular and short-lived and the female lays eggs immediately after its blood meal. The hibernating generation of adult anophelines hatches in the late summer when the temperature is too low for the sporogony to be completed. After mating they seek shelter in human dwellings to hibernate. During the main part of its adult life the female was inside a house together with humans. The hatched female may take its first blood meal outdoors but sporogony can only be completed indoors. Endemic malaria could thus only exist in Finland as an 'indoor' disease.

The spreading of malaria is best explained by movements of a large number of working people. Large working sites in Finland – the construction of the Saimaa canal in 1845–54, the lowering of lakes in the 19^th ^century, the building of the railway in 1862–71, etc. – brought temporary populations to uninfected areas. Some of the workers may have been carriers of *Plasmodium*. During the last weeks of the working season the adult anopheline females appeared and took their first blood meals and some of them became infected by *Plasmodium*. The working site was dispersed for the winter and the workers returned to their home villages and towns. The infected anophelines sought shelter in the houses of the local population, and the following spring the whole household was infected. A district physician reported from Ostrobothnia in 1845 and 1846 that malaria was common among carpenters, who had been working in the south of Finland [6]. The number of infected anophelines also grew significantly during the winter when uninfected females fed on infected humans. The cold season lasted for almost 8 months and since the incubation period for *P. vivax *could be 3–4 weeks in 18°C, it is probable that if the infection reached the household in September, all the members and the entire hibernating *Anopheles *population were infected by the following spring.

## Conclusion

The present study of the frequency of northern malaria cases and temperature data establishes the relation between malaria and climate. Endemic malaria existed exclusively as an indoor disease independently of climate. The temperature of the preceding summer regulated the amplitude of malaria cases. Infected people spread malaria into new regions, where mosquito populations transferred malaria locally. The mosquito-human interaction in human dwellings then maintained the introduced malaria in the new region.

*P. vivax *was the principal disease factor in Scandinavia and Finland. *P. falciparum *was not limited by winter conditions, but the lack of a summer dormant stage has been crucial in a cool maritime summer in north-western Europe. The continental summer is usually warm which could explain occasional outbreaks of *P. falciparum *malaria in northern Russia in the 1920's and 1930's. The final decline of malaria in the 20^th ^century in Finland is not explained by the current results. A statistical analysis of the changing demographic and ecological conditions is needed for a definitive explanation of that issue.

## Authors' contributions

LeH drafted the manuscript and collected the historical data. LaH contributed the temperature data and participated in the design of the study. KH conceived the study and participated in its design. All authors read and approved the final manuscript.

**Figure 7:**
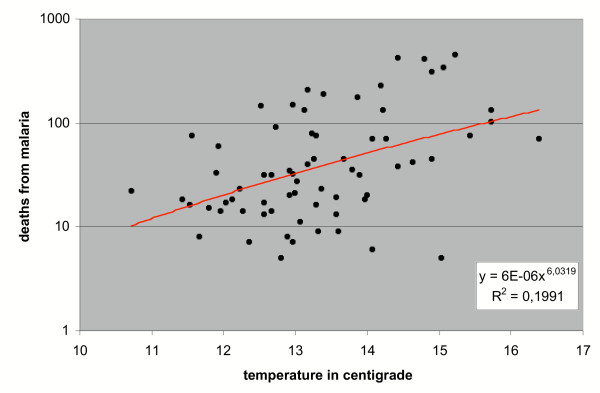
Deaths from malaria in relation to mean temperature of the preceding summer in Tornedalen 1803–1870.
